# Treacherous Pavements: Paving Slab Patterns Modify Intended Walking Directions

**DOI:** 10.1371/journal.pone.0130034

**Published:** 2015-06-11

**Authors:** Ute Leonards, John G. Fennell, Gaby Oliva, Alex Drake, David W. Redmill

**Affiliations:** School of Experimental Psychology, University of Bristol, Bristol, United Kingdom; Durham University, UNITED KINGDOM

## Abstract

Current understanding in locomotion research is that, for humans, navigating natural environments relies heavily on visual input; in contrast, walking on even ground in man-made obstacle and hazard-free environments is so highly automated that visual information derived from floor patterns should not affect locomotion and in particular have no impact on the direction of travel. The vision literature on motion perception would suggest otherwise; specifically that oblique floor patterns may induce substantial veering away from the intended direction of travel due to the so-called aperture problem. Here, we tested these contrasting predictions by letting participants walk over commonly encountered floor patterns (paving slabs) and investigating participants’ ability to walk “straight ahead” for different pattern orientations. We show that, depending on pattern orientation, participants veered considerably over the measured travel distance (up to 8% across trials), in line with predictions derived from the literature on motion perception. We argue that these findings are important to the study of locomotion, and, if also observed in real world environments, might have implications for architectural design.

## Introduction

Outdoor and indoor spaces in man-made environments are often paved (or tiled) with clearly visible grouting. People do not always walk in the direction of the grouting, and the dominant tile orientation often does not correspond to the direction of travel. Despite the undeniable importance of vision to the control of locomotion in humans [1] and evidence that a visual illusion painted on a flight of stairs to increase perceived step height leads to greater foot clearance [2], current biomechanical models would predict that on even obstacle- and hazard-free ground floor patterns should not matter, because walking in such an environment is heavily automated and overlearned (for a recent review see [3]). Reported research covering the impact of optic flow [4, 5] on the perception of self-motion and self-location in space would not predict too much impact of floor patterns either. Indeed, optic flow research tends to be based on specific objects or goals [6, 7], often in virtual environments [7, 8] where most influence should arise from the lateral visual periphery, not a comparatively narrow part of the lower visual field.

Nevertheless, the upper and lower visual fields are considered to statistically differ [9, 10], and the vision literature on motion perception leads to the assumption that floor patterns, in particular when found in corridors, will impact walking direction. More specifically, oblique floor patterns should induce substantial veering away from the intended direction of walking toward the closest floor pattern direction due to misperceived heading direction evoked by the aperture problem. The aperture problem refers to the inherent ambiguity of motion direction for a line seen through an aperture of limited size [11, 12]. Typically, we perceive motion normal to an edge, but perceived motion varies with the geometry of the aperture. In the human visual system, the aperture problem occurs whenever the detection of motion is carried out over a limited part of a scene such as in the lower visual field inherent for floor patterns. The effect can be easily visualised with a compelling motion illusion first described by Bressan and Vezzani [13]. In this illusion, visually tracking a dot that moves vertically between two stationary oblique lines makes the lines appear to move laterally. This illusion is strongest for lines tilted 10 to 30 degrees from vertical, and decreases rapidly as angles get larger. Translated into an environment in which a person is moving over diagonal stripes, these stripes should then also appear to shift laterally, inducing movement veer into the same direction. Most people living in modern, man-made environments will have walked across a paved/tiled surface, travelling in a direction diverging from the dominant visual orientation of that surface. Whether walking against the natural orientation of the floor pattern does impact a person’s ability to walk straight (in the intended direction) or nudges them in the direction of the pattern, consistent with the aperture problem, remains unknown.

**Fig 1:**
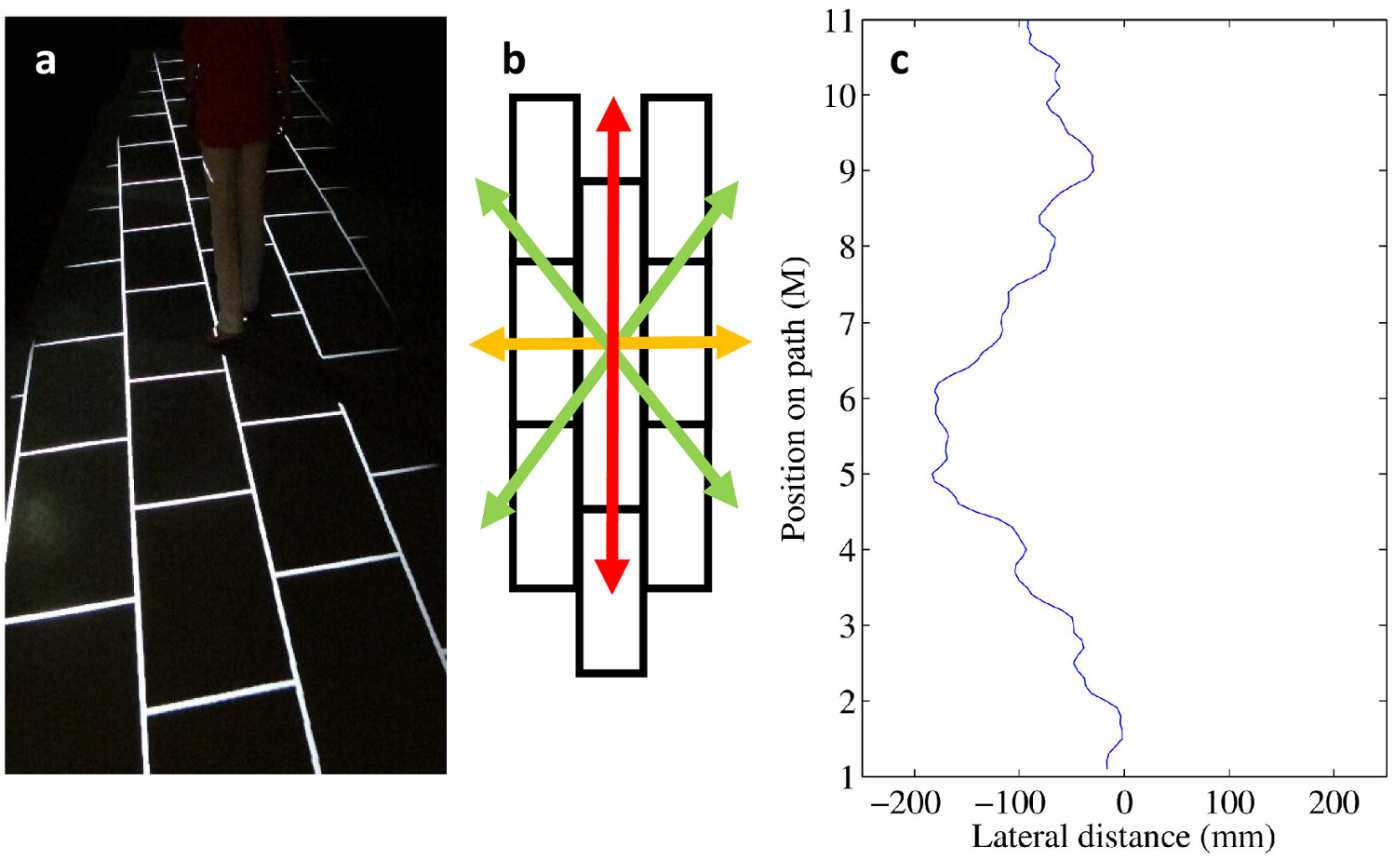
Examples of floor patterns and raw walking trajectories. Panel (a) shows a photograph with a possible floor projection used in the experiment. Panel (b) shows the 4 stimulus orientations included in floor tiling: red and yellow arrows mark basic single-component orientations (SCOs); the two green arrows mark bi-component orientations (BCOs). Panel (c) entails an example of a typical walking trajectory before spatial resampling and Principal Component Analysis: gait-related sway can easily be seen.

Using paving slab patterns similar to those seen on pavements, we investigated whether the walking-induced moving contours, formed by the tile shape and the grouting between the tiles or slabs, created a similar illusion to that described by Bressan and Vezzani [13], thus affecting people’s walking direction in an obstacle free environment. Note that pavement slab patterns contain basic single-component orientations (SCO) (see Fig 1b, orientation directions indicated by red and yellow arrows), consisting of high-luminance contrast lines (i.e. the grouting relative to the tiles) specific to a particular region within the pattern. They further contain bi-component orientations (BCO), spanning across the whole patterns and comprising multiple lines (see Fig 1b; orientation directions indicated by green arrows).

Participants were asked to walk over the floor of the Bristol Vision Institute (BVI) Movement laboratory at their normal walking speed while we systematically varied the orientation of the projected floor patterns from trial to trial (see Fig 1a). Although low-level lighting was used, windows were blacked out and black curtains covered the walls to minimize extraneous light and distractions, there were ample cues and reference points for vision-based walking: The ceiling was white, and the projectors provided sufficient light so that the camera and projector support systems consisting of vertical and horizontal scaffolding were clearly visible, providing stable visual reference frames throughout. Added to this, the projected path was 2 metres wide with clearly distinguishable edges and, during their walks, participants were asked to perform a rapid serial visual presentation (RSVP) task displayed on the far wall of the laboratory straight ahead of them, providing a clear target for heading direction. In other words, the visual environment necessary for the experimental setup would mitigate any effect of the floor pattern, similar to every-day walking conditions. Using 3D motion capture, it was investigated whether rotating the pattern orientations within the path (while keeping the outlines of the path constant), would influence participants’ walking trajectory; that is, we investigated whether different pattern orientations would induce lateral *veering*.

## Methods and Materials

Using a repeated measures design, healthy young adults walked over a projected walkway while performing a rapid serial visual presentation (RSVP) task projected onto the centre of the end wall of the laboratory, effectively at the end of the walkway. The RSVP task consisted of a random sequence of black and white outlined images of everyday objects (e.g., scissors, lamps, eggs, etc.) for which participants were asked to report whether the last image of a sequence had been included in the sequence before, or not. Each projected pattern of the walkway covered an area of 2 by 12 metres; and for each of the 64 walks performed per participant, one of 16 different paving slab patterns was projected in pseudo-random order. Floor patterns differed from each other by the orientation of their slabs (with a slab difference of 11.25° between individual conditions), but had otherwise identical outlines. Each floor pattern was repeated 4 times.

Floor patterns were displayed using a calibrated multi-projector (6) system (Optoma EW536) with an additional projector to display the RSVP task centrally onto the back wall. A multi-camera (12) Qualysis motion capture system with a spatial resolution of around 1mm^3^ and a recording frequency of 100Hz was used for recording the location of infrared reflectors that were attached to each participant (on the sternum, the talus and the heads of the 1st and the 5th metatarsal of each foot, both shoulders and knees, the midline of the waist and each lower hip joint; note that for the purpose of the current paper, only data of the sternum marker were analysed and no biomechanical modelling was performed).

The twenty-five participants comprised 7 males and 18 females (*M* age = 23.1, *SE* = 1.1). All participants reported normal or corrected-to-normal vision, and all provided their informed written consent prior to commencement of the experiment in line with the revised Declarations of Helsinki. The experiment was approved by the Faculty of Science Ethics Committee at the University of Bristol. Upon arrival, an explanation of the experiment was provided, and participants had the infrared reflectors attached to them. For each trial, participants placed their feet on two starting markers projected onto the floor and were asked to walk at natural speed straight along the pathway toward the other end of the lab, while performing the RSVP task ahead of them. On reaching the end of the pathway and providing a response to whether the final image had been seen previously, participants were instructed to return to the starting points after a brief pause. Participants’ verbal responses to the RSVP task were recorded and found to be over 80% correct in all participants. At the outset, participants were given a practice trial so that they felt confident performing the RSVP task while walking down the laboratory. After completion of the experiment, markers were removed and participants were debriefed formally.

### Data Analysis

As we were interested in lateral veering during walking, we restricted our analysis to the sternum marker data only. First, the sternum marker was labelled for each trial using Auto Identification Models from the Qualysis Track Manager software; then, we deleted the first and the last meter of the recorded trajectory to avoid noise induced by the start of walking, and by participants starting to turn when reaching the end of the projected walkway. This left us with 10m of recorded data per walk (see Fig 1c for a typical example path). Next, data were spatially resampled (i.e. 100 equidistant samples per 10m path), before we performed a Principal Components Analysis (PCA), because such an approach provides a small number of values that describe each walking trajectory well without being sensitive to gait-related lateral sway present in most participants when stepping from one foot onto the other, and which is capable of being statistically analysed.

PCA finds a set of principal component coefficients, or a basis function, that ‘shoe horns’ as much information as possible, measured by variance, into the early principal components. Often, the number of principal components needed for the majority of variance is small; in the present case, the first principal component accounts for 73.4% of the variance in the trajectories. Multiplying the coefficients (in this case a vector of values for the first principal component coefficient) by the path data recorded for each participant provides a set of scores, the actual ‘principal components’.

Based on the high percentage of variance accounted for by the first principal component in our data, we decided to restrict our analysis to this first principal component. This means that there is only one number (the score) that summarises each trajectory across the 100 spatially sampled points (10 samples per meter of the 10m walkway analysed) and makes carrying out statistical tests, such as t-tests and ANOVAs, straight forward. Since it was observed that participants ‘began slowly’, with greater eccentricity being apparent towards the middle of the pathway, and often a correction towards the end, but with great intra- and inter-individual variability when the maximal eccentricity was reached within a given walk, the outcome of the first principal component is equivalent to the cumulative (and direction) of lateral veer irrespective of where exactly in the pathway it occurs.

As we were not interested in absolute veer, but the relative veer for oblique orientations compared to our baseline conditions, we first defined our baseline as the average between participants’ first PCA for floor patterns of 90 and 0 degrees, and then calculated the difference scores for first PCAs for each orientation relative to this baseline.

## Results

**Fig 2:**
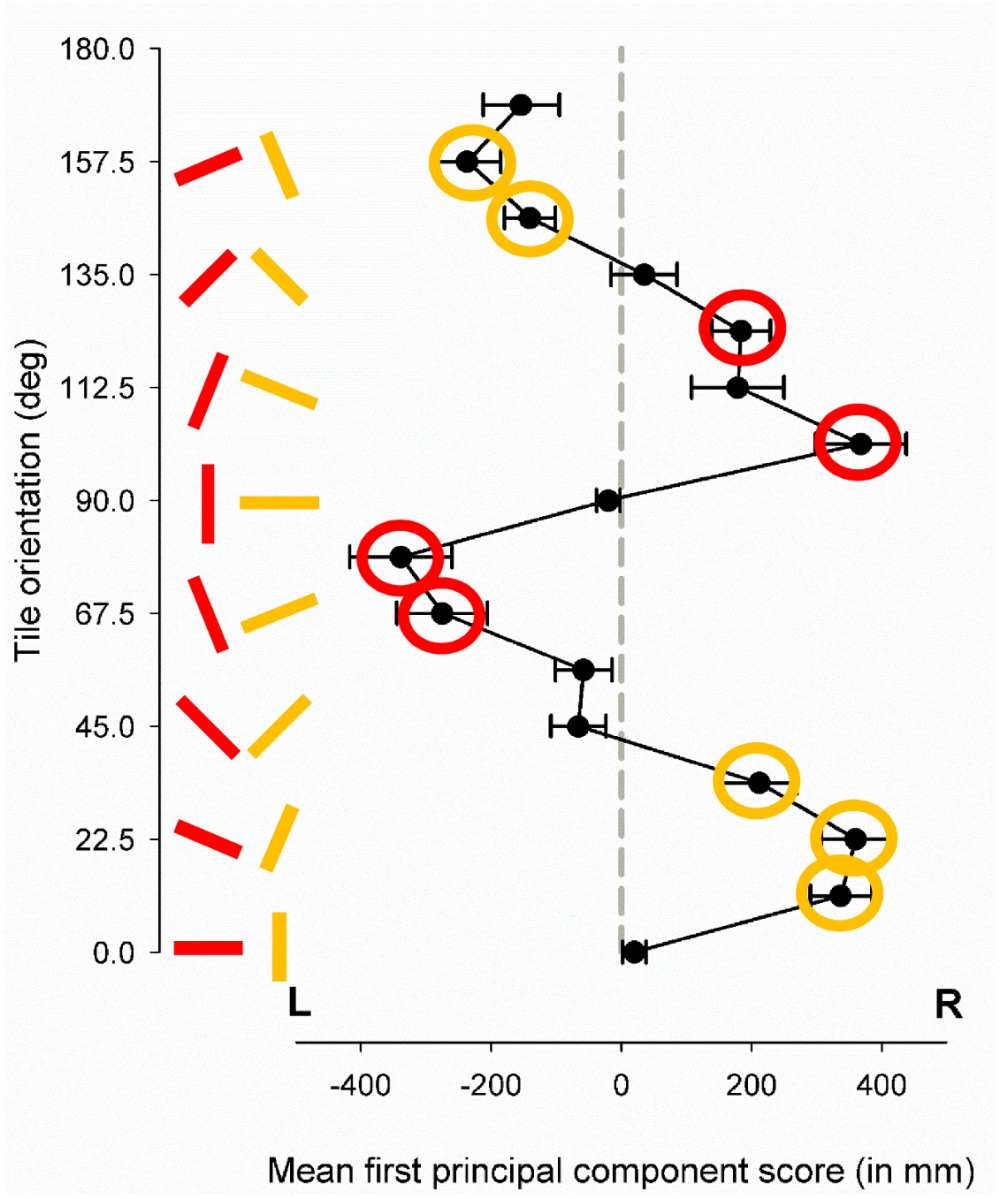
The orientation of floor tiles can substantially direct people’s walking trajectories away from the intended walking direction. Group average (±1SEM) of maximum lateral veer per walk over 10m (expressed through the first principal component score) for each of the different floor tile orientations, relative to baseline (average lateral veer across floor pattern orientations of 90 and 0 degrees). The red and yellow bars show schematically the major single component tile orientations (SCOs) as expressed in Fig 1(b): 90 degrees correspond to the long SCO in the direction of travel; 0 degrees correspond to the long SCO orthogonal to the direction of travel. Participants veered to the left or to the right as compared to the baseline (average veer across 90 and 0 degrees; see grey dotted reference line), in dependence of which of the two basic SCOs was closer to the direction of travel. Veer significantly different from the baseline is marked by circles in the colour of their respective SCO (p<.003, corrected for multiple comparisons). Bi-component orientations did not seem to attract lateral veer toward them.

We found that for some paths, participants’ general trajectories *veered off* significantly from our straight ahead baseline (see Fig 2). A one-way repeated measures ANOVA was conducted to compare the effect of tile orientation on veering, using the first principal component as dependent variables. Mauchley’s test revealed that the assumption of sphericity had been violated, χ^2^(119) = 205.7,p<.001; therefore, degrees of freedom were corrected using Huynh-Feldt estimates of sphericity (ε = .405). There was a highly significant effect of floor pattern orientation on the extent of veering, F(6.8,139.9) = 21.733, p<.001, partial η^2^ = .486, observed power = 1.00. Bonferroni-corrected *post-hoc* pairwise comparisons confirmed that the veer induced by the two baseline conditions of 0° and 90° did not differ from each other (mean difference = 40mm; p>.99). In addition, the two oblique orientations of 45° and 135° did not differ significantly from each other (mean difference = -100mm; p>.99), nor did they differ significantly from the two baseline orientations (mean differences between -85mm and +54mm, p>.99), indicating that the two SCOs balanced each other out. More importantly, one-sample t-tests against 0, corrected for multiple comparisons, revealed that participant veered significantly to the right as compared to averaged baseline for floor orientations of 11.25°, 22.5°, 33.75°, 101.25° and 123.75° (t>3.7; p<.003), and significantly to the left for floor orientations of 67.5°, 78.75°, 146.25°, and 157.5° (t<-3.6; p<.003). Veering was thus along the direction of the basic SOC that was closest to straight ahead, with oblique as compared to baseline floor patterns inducing lateral veering of up to 370mm over the measured travel distance of 10 meters. Taking the extent of the induced veer as a measure of strength, it is easy to see from Fig 2 that the illusion was strongest when the grouting lines were between 11.25 and 22.5 degrees tilted relative to straight ahead. Interestingly, this applied whether it was the 'long' (Fig 2 red orientation bars) or the 'short' (Fig 2, yellow orientation bars) grouting lines that were at these angles, with veering in the direction of the lines.

## Discussion

With a pattern of results consistent with those of Bressan and Vezzani’s visual motion illusion [13], we found that participants veered substantially away from straight ahead when walking over a patterned floor with oblique pattern orientations. We propose that the veering observed here is due to the aperture problem in motion perception [11] in the relatively restricted lower visual field, leading people to misperceive their own lateral location in space while traversing the walkway.

Before being able to speculate about possible implications of such a finding on everyday walking in man-made environments, it is important to consider the generalizability of our findings. Indeed, our experimental design included walking in a comparably narrow corridor (2m wide) under reduced lighting conditions, reversed-contrast floor patterns of comparably high contrast (white/light grey “grouting” with black tiles), and one tile size only; also, participants walked while performing a rapid serial visual presentation task that kept their gaze on the far wall of the lab that they were approaching and thus provided them with clear heading directions and a clear focus of attention, away from the actual walking task. It seems unlikely that this particular experimental setup could be solely responsible for the veering observed: veering happened only for certain (oblique) pattern orientations, though experimental conditions such as corridor width, lighting and task did not differ between different pattern orientations. More importantly, the *direction* of veering consistently followed one of the two single-component orientations present in the patterns, namely that closest to the intended direction of walking. We have no evidence that bi-component orientations, though clearly visible under some pattern orientations but not others, affected people’s walk. Such observations provide evidence against the possibility that participants consciously “latched on to” floor orientations, because they thought that this was what was required. Further evidence against such demand characteristics being the cause of our findings is that none of our participants was able to deduce the goal of the experiment or to describe the manipulations we had tested when asked at the end of the session; i.e. nobody had noted that floor pattern orientations had been varied.

However, we cannot exclude that the strength of the observed lateral veer (up to 40 cm over the 10 meter length of a single walk; thus up to 80 cm difference between different patterns) depended on experimental conditions. In particular, lateral veer might be more or less pronounced depending on the relationship between spatial frequencies chosen for the patterns and the corridor width [14], or on the chosen combination of spatial frequency and orientation [15]. More importantly, the degree of veer might vary according to whether participants concentrate on a clear target ahead of them (as in our setup) or look around freely during the actual walk, possibly even directing their gaze onto the floor. Indeed, preliminary data of a study in which cognitive load was specifically manipulated [16], revealed increased veer for walks under higher cognitive load. Note that in our experiment we can exclude systematic changes in cognitive load as explanation for increased veer as participants’ performance for the RSVP task did not differ for different floor orientations. However, future experiments should systematically explore how the strength of veer depends on the exact environmental and task conditions, including investigation of real world settings.

In conclusion, under hazard- and obstacle-free walking conditions, visual information on the floor impacts on the control of walking, in particular when gaze is directed straight ahead toward a clearly visible target. Since walking was affected by merely changing pattern orientations on the floor, walking direction seems far more shaped by our environment than would be predicted from current locomotion models based on optical flow [6, 7]. Future investigations are clearly asked for that help us to understand the potential consequences patterned paving/flooring may have for walking trajectories and, ultimately, for the person walking. Questions to be explored range from whether veer leads to imbalances within the locomotion system that could potentially increase the risk of falls, in particular in older people [17], to whether floor patterns could be used to steer people away from dangerous areas.
